# Formation of ferromagnetic Co–H–Co complex and spin-polarized conduction band in Co-doped ZnO

**DOI:** 10.1038/s41598-017-11078-3

**Published:** 2017-09-11

**Authors:** Seunghun Lee, Ji Hun Park, Bum-Su Kim, Deok-Yong Cho, Yong Nam Choi, Tae-Woo Lee, Won-Kyung Kim, Doukyun Kim, Chae Ryong Cho, Chikako Moriyoshi, Chul Hong Park, Yoshihiro Kuroiwa, Se-Young Jeong

**Affiliations:** 1 Department of Materials Science and Engineering, University of Maryland, College Park, Maryland 20742 USA; 2 Department of Cogno-Mechatronics Engineering, Pusan National University, Miryang, 50463 Republic of Korea; 3 IPIT and Department of Physics, Chonbuk National University, Jeonju, 54896 Republic of Korea; 4 Neutron Science Center, Korea Atomic Energy Research Institute, Daejeon, 34057 Republic of Korea; 5 KAIST Analysis center for Research Advancement, Daejeon, 34141 Republic of Korea; 6 Department of Physics, Pusan National University, Busan, 46241 Republic of Korea; 7 Department of Nanoenergy Engineering, Pusan National University, Busan, 46241 Republic of Korea; 8 Department of Physical Science, Hiroshima University, Higashi-Hiroshima, 739-8526 Japan; 9 Department of Physics Education, Pusan National University, Busan, 46241 Republic of Korea

## Abstract

Magnetic oxide semiconductors with wide band gaps have promising spintronic applications, especially in the case of magneto-optic devices. Co-doped ZnO (ZnCoO) has been considered for these applications, but the origin of its ferromagnetism has been controversial for several decades and no substantial progress for a practical application has been made to date. In this paper, we present direct evidence of hydrogen-mediated ferromagnetism and spin polarization in the conduction band of ZnCoO. Electron density mapping reveals the formation of Co–H–Co, in agreement with theoretical predictions. Electron spin resonance measurement elucidates the ferromagnetic nature of ZnCoO by the formation of Co–H–Co. We provide evidence from magnetic circular dichroism measurements supporting the hypothesis that Co–H–Co contributes to the spin polarization of the conduction band of hydrogen-doped ZnCoO.

## Introduction

Over the past few decades, despite numerous theoretical and experimental investigations, the origin of ferromagnetism in Co-doped ZnO (ZnCoO) has remained a mystery. This has been an obstacle to further progress in its applications. In most previous studies, researchers attempted to explain the ferromagnetism and spin-dependent phenomena observed in ZnCoO in terms of magnetic clusters^1–4^, carrier mediation^5, 6^, or magnetic polarons based on intrinsic defects^7–9^. However, none of these can clearly explain the inconsistent results reported. Therefore, many alternative models of ferromagnetism in ZnCoO have been proposed. The occurrence of ferromagnetism and the strength of the magnetization appear to be strongly dependent on the sample fabrication conditions and environment. Several researchers have even suggested that pure ZnCoO^10–14^ is not ferromagnetic. These results have raised doubts concerning the possible effects of unintentional defects and/or impurities, which are likely to affect the magnetic properties of ZnCoO^15–28^.

Hydrogen is an inevitable impurity that is easily incorporated into ZnO-based materials and consequently gives rise to intrinsic n-type conductivity^7, 29^. Several theoretical models have been developed in which the effects of hydrogen impurities cause ferromagnetism^30–35^. The correlation between magnetism and hydrogen content has been demonstrated experimentally^36–40^. However, no conclusive evidence has been presented that hydrogen causes ferromagnetism in ZnCoO, and there remain doubts about whether the ferromagnetism is extrinsic. This would be the case if it was caused by a metallic Co phase or the oxygen vacancy effects, which may occur when chemical reduction is accompanied by hydrogen treatment^17, 41^.

In this study, we present evidence for hydrogen-mediated ferromagnetism and spin-polarized conduction band in ZnCoO. Electron density distribution constructed from synchrotron x-ray diffraction (XRD) patterns and the maximum entropy method visualizes the formation of a Co–H–Co complex that is theoretically predicted to give rise to the ferromagnetism of ZnCoO. The results of electron spin resonance (ESR) measurement support the hypothesis that the antiferromagnetic ordering of nearest-neighbored Co–Co is converted into a ferromagnetic ordering by the formation of Co–H–Co. Magnetic circular dichroism measurements reveal the evolution of the spin-polarized conduction band correlated with the hydrogen content. We suggest that the Co–H–Co complex contributes to the spin polarization of the conduction band. The results of structural analyses with synchrotron XRD, X-ray absorption spectroscopy (XAS), and transmission electron microscopy (TEM) exclude other possible extrinsic sources of ferromagnetism and spin-dependent band structures in ZnCoO.

## Results

### Exclusion of Unintentional Effects

**Figure 1:**
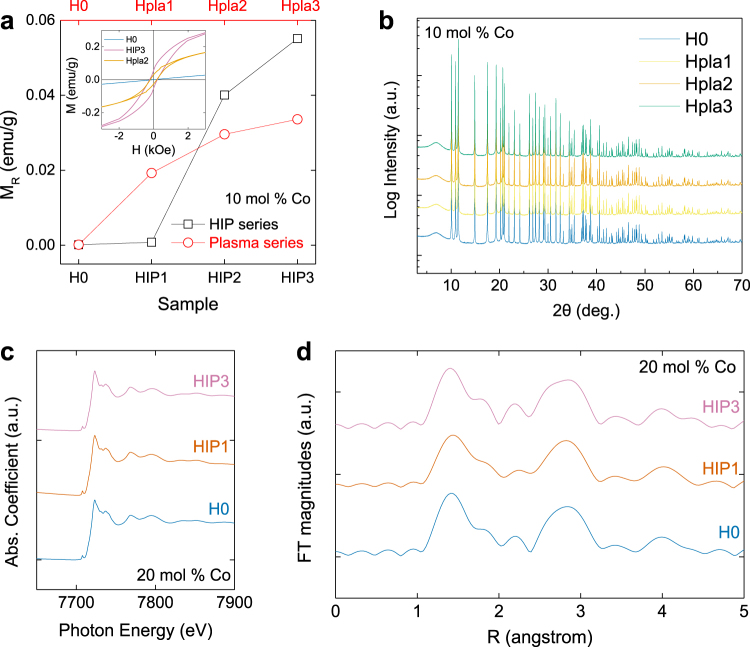
(**a**) Remnant magnetization (M_R_) of ZnCoO powders with varying hydrogen injection conditions (both HIP- and plasma-series) measured at room temperature. The inset shows field-dependent magnetization curves of H0, HIP3, and Hpla2. (**b**) Synchrotron X-ray diffraction patterns of ZnCoO powders (plasma-series) obtained at 95 K. (**c**) X-ray absorption near-edge structure (XANES) and (**d**) extended X-ray absorption fine structure (EXAFS) spectra of ZnCoO powders (HIP-series). Note that, in the X-ray absorption (XAS) experiments (XANES and EXAFS), we used ZnCoO powders containing 20 mol % Co.

To exclude the effects of secondary phases that might cause ferromagnetism-related features, we carried out careful structural analysis employing synchrotron radiation XRD and XAS experiments. We prepared 10 and 20 mol % Co-doped ZnO powder samples with different hydrogen concentrations. We obtained different hydrogen concentrations using different hydrogen treatment methods and conditions. Details regarding the sample preparation can be found in the Methods section. H0 denotes a pristine ZnCoO sample (*i.e*., without intentional hydrogen treatment), and the HIP (HIP-series) and Hpla (plasma-series) indicate hydrogen-treated samples. The treatments are hot isostatic press and plasma treatment, respectively. The number in each label indicates the relative hydrogen content that was determined using secondary ion mass spectroscopy. Thus, the amount of hydrogen increases as the number increases. Figure 1a shows how the remnant magnetization (M_R_) of the ZnCoO samples varies with the hydrogen treatment conditions. As the hydrogen content increases, M_R_ gradually increases. This demonstrates that the evolution of ferromagnetism correlates with the amount of hydrogen in the sample. The inset of the figure shows the representative field-dependent magnetization curves of H0, HIP3, and Hpla2. A ferromagnetic hysteresis loop can clearly be observed in the ZnCoO samples with injected hydrogen.

Figure 1b shows the synchrotron X-ray diffraction patterns of the ZnCoO samples (Hpla-series). The diffraction patterns of each sample are almost identical. According to the Rietveld refinement, no secondary phase other than wurtzite ZnO was present. The reliability factor (*R*_*F*_) was below 0.8% for each sample (H0: *R*_*F*_ = 0.597%, Hpla1: *R*_*F*_ = 0.736%, Hpla2: *R*_*F*_ = 0.629%, and Hpla3: *R*_*F*_ = 0.669%). The structural parameters (*e.g*., the lattice constant, bond length, and bond angle) of the basal plane obtained from the Rietveld refinement differed by less than 0.01% between the samples. The oxygen occupancy was also found to be almost identical for each sample (see Supplementary Information). This supports the hypothesis that the hydrogen plasma treatment does not induce any significant structural changes that could cause magnetism, such as the introduction of defects and/or secondary species.

Figure 1c shows the Co K-edge X-ray absorption near-edge structure (XANES) spectra of the ZnCoO powders (HIP-series). The main features at 7723 eV, as well as the higher energy features, reflect the dipole transitions from the Co 1 s core level to the p-continuum states. The overall intensities and line shapes of the XANES features are mostly determined by the local structures near the central Co^2+^ ions. All spectra appear to be superimposable; the difference between the XANES spectra of H0 and HIP3 is only 0.212%, which indicates that hydrogen injection does not significantly change the electronic structure of Co^2+^ ions. In addition, each spectrum in Fig. 1c shows the signature of 1 s → 3d pre-edge absorption of Co^2+^ at 7709 eV. This is a well-known electronic transition for substitutional Co^2+^ in ZnO^16, 42^. This result indicates that Co^2+^ ions are well substituted into Zn sites in the ZnCoO sample.

The Fourier transform (FT) was processed based on a *k*-weighted XAFS modulation, where *k* is the final-state electron momentum. The magnitudes of the FTs of the extended X-ray absorption fine structures (EXAFS) of each sample in the range of k = 2–11 Å^−1^ are shown as a function of the phase-uncorrected interatomic distance R in Fig. 1d. The peaks near R = 1.4 Å indicate Co–O coordination, and those near 2.8 Å correspond to Co–Co next-nearest-neighbor coordination in the cobalt oxides. As shown in Fig. 1d, the EXAFS data also suggest that there is no significant change in the local structure around the Co ions. Apart from fluctuations of the main oxide peaks, there are no clear features near R = 2.5 Å, which would correspond to the Co–Co distance of metallic cobalt. We calculated the Co–O and Co–Co bond lengths from the EXAFS fittings and found them to be ~ 1.9 Å and ~ 3.2 Å, respectively. These lengths are consistent with the values for cobalt oxides rather than cobalt metals. These findings indicate that the formation of Co metal clusters or crystalline defects is not traceable and thus negligible^16, 42, 43^. Consequently, it is possible to exclude unintentional effects as the source of ferromagnetic features.

### Evidence of the Formation of Co–H–Co Complexes

**Figure 2:**
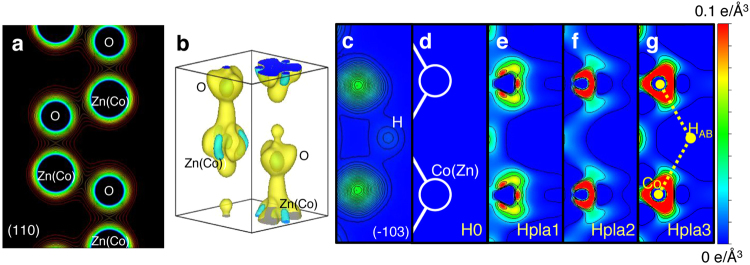
(**a**) The electron density distribution of the sample without injected hydrogen (H0) in the (110) plane. This was constructed using the maximum entropy method (MEM). The electron density of Hpla3 (with hydrogen injection) differs slightly. The contours are from 0.3 e/Å^3^ to 3.0 e/Å^3^, in steps of 0.1 e/Å^3^. (**b**) The electron density distribution image obtained by subtracting the electron density distribution of H0 from that of Hpla3. The lattice constants of the unit cell of wurtzite ZnCoO are a = 3.25 Å and c = 5.20 Å. The isosurface is at ±0.02 e/Å^3^, where the yellow area corresponds to increased electron density following hydrogen injection, and the blue area indicates a decrease in the electron density following hydrogen injection. (**c**) The theoretical electron density distribution of the Co–H–Co complex in the (−103) plane, in which a Co dimer and a H atom are theoretically predicted to exist. (**d**–**g**) The electron density maps of H0, Hpla1, Hpla2, and Hpla3 in the (−103) plane were obtained by subtracting the electron density map of H0. Thus, the H0 density map is empty.

To investigate how hydrogen interacts with ZnCoO, we constructed electron density maps of ZnCoO samples (plasma-series) from the synchrotron XRD patterns using the maximum entropy method. Figure 2a shows the electron density map of H0 in the (110) plane. In principle, X-ray diffraction data show averaged structural information; therefore, if the structural parameters of hydrogen-doped samples are similar, it is difficult to observe changes caused by hydrogen doping in bare electron density maps. We were not able to see any apparent differences in the bare electron density maps of the ZnCoO samples. Thus, we attempted to observe subtle changes in the electron density by subtracting the electron density of the reference samples (H0) from the electron density of the Hpla1, Hpla2, and Hpla3 samples.

Figure 2b shows the three-dimensional electron density distribution obtained by subtracting the electron density distribution of H0 from that of Hpla3. Thus, this distribution shows the change in electron densities caused by the intentionally introduced hydrogen. The most prominent change is the increase in the electron density at the bond center (BC) and antibond (AB) sites along the c-axis. These sites are known to be the most stable hydrogen positions in ZnO^29^. The difference in the electron density around each atomic site is caused by the electronic interaction between hydrogen and the adjacent atoms.

To investigate further, we examined the electron density in the (−103) plane. The Co dimers and anti-bonding hydrogen at the hexagonal center (H_AB_) are theoretically predicted to be located in this plane and to initiate ferromagnetism by the formation of Co–H–Co^30^. Figure 2c shows the theoretical electron density of the Co–H–Co complex in the (−103) plane, and Fig. 2d–g show the differences in the electron density maps between H0, Hpla1, Hpla2, and Hpla3 and H0. Therefore, H0 is used as a reference and Fig. 2d shows an empty density map. We are not able to see the electron density corresponding to the hydrogen sites due to the small scattering factor of hydrogen. Instead, we can clearly see a gradual change in the electron density distribution near the cation sites (*i.e*., Zn or Co ion). The electron density changes significantly as the hydrogen content increases. The electron density at the atomic center of Zn(Co) decreases, while in the vicinity of Zn(Co) it changes significantly, moving towards the H_AB_ sites whilst maintaining threefold symmetry along the c-axis. This indicates that Zn(Co) ions move towards the H_AB_ site, and this displacement can occur in three equivalently possible ways. It is worth noting that no such displacement is observed in hydrogen-doped pure ZnO (not shown here). Therefore, we can ascribe the atomic displacement to the interaction between the Co atoms and hydrogen. We have also theoretically analyzed the energetic stability of other possible H_AB_ sites (i.e., at Zn–Zn or Zn–Co pairs), but the H_AB_ sites at the Co–Co dimer were found to be the most stable, and hence these sites would be preferentially occupied by H atoms.

**Figure 3:**
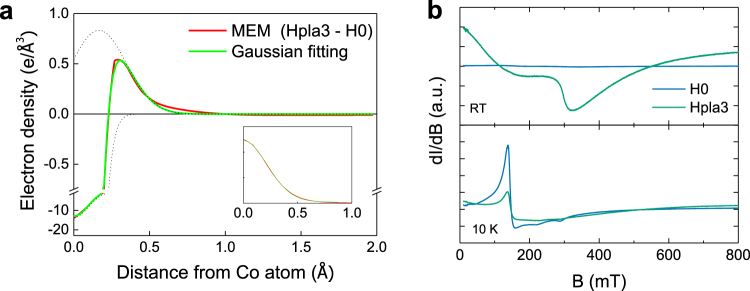
(**a**) Electron density line profile of Hpla3–H0 along the Co_Zn_–H_AB_ bond (i.e., the yellow dotted line in Fig. 2g). The inset shows the electron density line profile of a Co_Zn_ atom obtained from H0. (**b**) Electron spin resonance (ESR) spectra of H0 and Hpla3. The features of the ESR spectra measured at 10 K and 300 K originate from paramagnetic and ferromagnetic resonance of the Co ions, respectively.

We estimated the displacement of the Co atoms towards the H_AB_ site from the line profile of the electron density. This is indicated by the yellow dotted line in Fig. 2g. Figure 3a shows the line profile corresponding to this line. We first used the Gaussian function to model the line profile of H0, as shown in the inset of Fig. 3. Two model functions (one positive and one negative) were used to fit the line profile. The negative function corresponds to the electron density of the Co atoms before the hydrogen treatment. The positive function represents the displacement of the Co atoms induced by hydrogen incorporation. By finding the position of the peak of the positive Gaussian model function, we found the displacement to be 0.166 Å. This value is in good agreement with the *ab initio* theoretical estimate of the displacement caused by the formation of a Co–H–Co complex, which is 0.187 Å.

Theoretically, Co ions in Co-Co dimer are antiferromagnetically coupled. The formation of a Co–H–Co complex makes them ferromagnetic^30^. To investigate how the formation of Co–H–Co complexes affects the spin states of the Co ions, we performed ESR measurements of the H0 and Hpla3 samples. Figure 3b shows the ESR spectra of H0 and Hpla3 measured at 10 K and 300 K. The ESR intensity is the first derivative of the absorption with respect to field and normalized by the sample mass. The ESR spectra measured at 10 K exhibit typical electron paramagnetic resonance (EPR) signals for Co^2+^ (*g ~* 2.2 and *g* ~ 4.5) substituted Zn^2+^ sites^44, 45^. The EPR signal decreases in hydrogen-doped ZnCoO (Hpla3). As the EPR intensity is proportional to the number of paramagnetic and antiferromagnetic-coupled Co ions, the decrease in the EPR signal indicates that the magnetic state of some portion of the originally paramagnetic and/or antiferromagnetic Co ions has changed as a result of the hydrogen doping. Hpla3 exhibits a broad ESR signal at 300 K. This signal is caused by ferromagnetic resonance (FMR)^46^. The broadness can be attributed to the random crystalline orientation and large Gilbert damping parameter, which is caused by the large particle size^47–50^. The ESR results support the theoretical prediction that hydrogen causes antiferromagnetic Co-Co dimers to be converted into ferromagnetic Co–H–Co complexes.

### Spin Polarization of the Conduction Band of ZnCoO

One of the key issues in magnetic semiconductor research is proving the existence of spin-polarized carriers as the result of interactions between the conduction band and magnetic impurities (*i.e*., *s-d* exchange interaction for transition metal-doped ZnO). We have reported the anomalous Hall effect in hydrogen-doped ZnCoO as the evidence of the presence of spin-polarized carriers^51, 52^. However, the anomalous Hall effect does not fully account for the spin polarization of the conduction band. To clarify this issue, we used magnetic circular dichroism (MCD) measurements to investigate the spin-polarized band structures. MCD is caused by the differences in the absorption of left and right circularly polarized light in spin-dependent band structures. MCD has been used to investigate the spin-dependent band structure of various magnetic semiconducting materials^53–56^. Using MCD measurement, we examined the evolution of the spin-dependent band structure of ZnCoO as the hydrogen content increases. For the MCD measurements, we used plasma-treated ZnCoO thin films with varying amounts of hydrogen (plasma-series). Details regarding the sample preparation can be found in the Methods section. We note that H0 thin film in the MCD study was prepared by subjecting a pristine ZnCoO thin film to a hydrogen plasma treatment at 20 W in order to increase the carrier concentration of a pristine ZnCoO (~10^16^/cm^3^).

**Figure 4:**
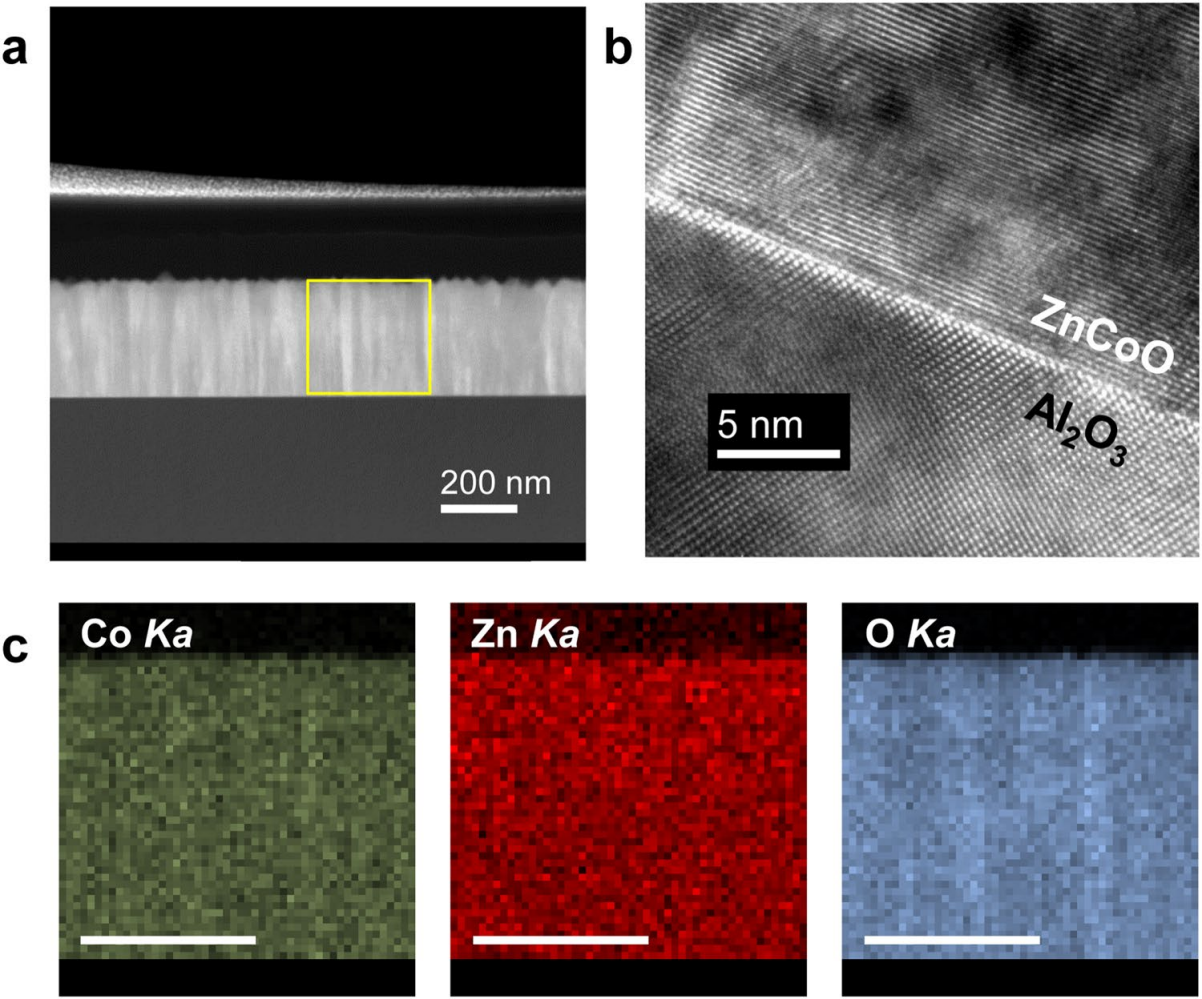
(**a**) Low-magnification image of hydrogen-doped ZnCoO thin film (Hpla3). (**b**) High-resolution transmission electron microscopy (TEM) image of the interface between the Hpla3 thin film and the Al_2_O_3_ substrate. (**c**) Energy-dispersive spectroscopy (EDS) compositional mappings of the Co, Zn, and O atoms in the yellow square area in (**a**) (scale bar indicates 200 nm).

The XRD measurements of the ZnCoO thin films with injected hydrogen indicated no trace of structural deformation within the resolution limit (see Supplementary Information). For more careful structural analysis, we analyzed Hpla3 using high-resolution transmission electron microscopy (TEM). Figure 4a shows the cross-sectional magnified image of Hpla3 and the yellow square represents the region used for energy-dispersive spectroscopy (EDS) compositional mapping (Fig. 4c). The EDS compositional mapping reveals that each atom (Zn, Co, and O) is homogenously distributed across the entire region. No signature indicating the aggregation of Co ions is observed. Figure 4b shows a high-resolution TEM image near the interface between the Hpla3 thin film and the Al_2_O_3_ substrate. No evidence of extra phases, such as columnar structures is observed, which indicates the absence of Co-metal clusters or Co-rich wurtzite clusters^39^. Although we do not show them here, atomic force microscopy measurements of the surface roughness revealed that the surface of Hpla3 (rms ~1 nm) was extremely smooth. All of the structural analyses suggest that no structural degradation occurs as a result of hydrogen injection.

Prior to the MCD measurement, we measured the ultraviolet–visible (UV-vis) absorption spectrum. This enabled us to evaluate whether the hydrogen treatment caused changes in the optical properties and band structure of the ZnCoO thin films (see Supplementary Information). The absorption spectra of the thin films were almost identical, with no reductions or shifts in the features corresponding to the Co^2+^ d-d^*^ transition. This indicates that the valence state of the Co is not affected by hydrogen treatment^57, 58^. We also derived the optical band gap energy of each thin film using a Tauc plot. All of the thin films had the same band gap energy of 2.89 ± 0.01 eV.

**Figure 5:**
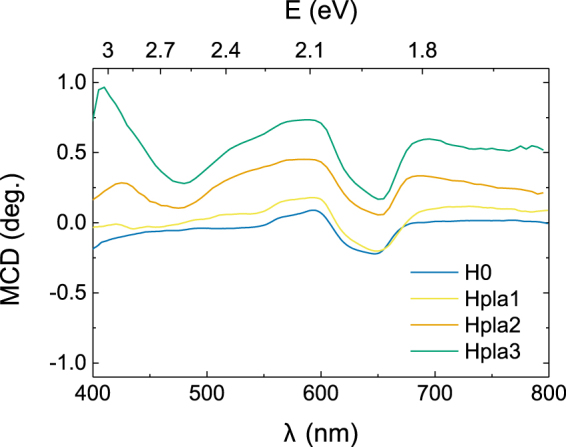
Magnetic circular dichroism (MCD) spectra of ZnCoO thin films (plasma-series). The MCD spectra were measured at a temperature of T = 10 K and magnetic field of B = 0.4 T.

Figure 5 shows MCD spectra of the ZnCoO thin films. The details of the MCD measurements are described in the Methods section. All of the spectra have the same general features at ~2 eV: a spin-orbit split 4A_2_ → 4T_1_ (P) ligand-field band of Co^2+^ substituted into a Zn^2+^ site in the wurtzite ZnO structure^58^. However, two distinguishable features are observable as a result of the hydrogen treatment: 1) a very broad positive signal is superimposed over the entire range and 2) the MCD intensity at 430 nm increases significantly. These features directly indicate the evolution of the spin-dependent band structure resulting from hydrogen doping. In particular, the energy corresponding to a wavelength of ~ 430 nm corresponds to the band gap energy of ZnCoO and the MCD features around 430 nm thus imply that the conduction band is spin-polarized. The intensity of the MCD near 430 nm increases with increasing hydrogen content, which indicates that the degree of spin polarization is correlated with the amount of hydrogen. This feature and the spin polarization of the ZnCoO conduction band have been attributed to a spin-split donor impurity band near the conduction band minimum (CBM)^46, 59^. As hydrogen creates shallow donor levels near the CBM, the donor impurity model may appear to account for this result. However, we have observed the absence of ferromagnetism in Al-doped ZnCoO (carrier concentration ~ 10^19^/cm^3^) with a higher oxygen deficiency than pure ZnCoO^52, 60^. This indicates that the donor impurity or oxygen defect model does not satisfactorily explain the ferromagnetism and spin polarization of ZnCoO^61^. Additionally, every sample used in the MCD measurement exhibited the same order of carrier concentration (~10^18^ /cm^3^, see Supplementary Information). Hence, the spin polarization of the conduction band is strongly correlated with the formation of a Co–H–Co complex, since this cannot be explained by the donor impurity model itself. To support this idea, we have theoretically investigated the spin-dependent density of state (DOS) of ZnCoO and Co-H-Co. The possible contributions of Co–H–Co levels onto the spin polarization are discussed in detail in the Supplementary information.

## Conclusion

In summary, injecting hydrogen into ZnCoO initially causes a shallow donor level, and then subsequently forms Co–H–Co complexes. We provide the first direct evidence of the formation of Co–H–Co complexes by electron density mappings constructed by synchrotron XRD patterns and the MEM. The emergence of robust ferromagnetism and spin-dependent band structure is strongly correlated with the formation of Co–H–Co complexes as well as hydrogen content. This work serves experimental insights into the contribution of hydrogen on the physical properties of ZnCoO.

## Methods

### Characterization

Synchrotron X-ray diffraction (XRD) patterns were collected at the BL02B2 beam-line of SPring-8 (Japan). This was equipped with a large Debye-Scherrer camera to enable precise measurements of the diffraction patterns from powder samples^62, 63^. The electron densities were analyzed using 122 structure factors obtained from the synchrotron XRD data via the visualization program VESTA^64^. The calculations were carried out using the ENIGMA software package^65, 66^, with 66 × 66 × 104 pixels. A reliability factor of *R*_*F*_ < 0.8% was obtained for all samples, which is small enough to trace changes in the electron density following hydrogen incorporation and consequent electronic interactions with neighboring Co atoms^67, 68^.

The XAS was performed in transmission mode at the 10 C beamline in the Pohang Light Source (PLS). We analyzed the XAFS using the UWXAFS package. For ESR measurement, we used a Fourier transform electron spin resonance (ESR) spectrometer (JEOL, JES PX2300). The field dependent magnetization was measured using a vibrating sample magnetometer (VSM) in physical properties measurement system (Quantum Design, Inc.).

XRD measurement for the thin films was performed using θ/2θ geometry on a PANalytical Empyrean Series 2 instrument equipped with a Cu–Kα source (40 kV, 30 mA). Diffraction data were collected at diffraction angles, 2θ, from 20 to 80°. Atomic force microscopy (AFM) and magnetic force microscopy measurements were carried out using a commercial AFM system (XE-100, Park Systems, Inc.). High-resolution transmission electron microscopy (HR-TEM) measurements were performed using a Tecnai TF30 ST (FEI) system operating at an acceleration voltage of 300 kV; a focused ion beam (Helios, 450 F1) was used for TEM sample preparation.

MCD measurements were carried out by means of the polarization modulation produced by a photo-elastic modulator. The samples were placed in an optical cryostat equipped with a 0.5-T electromagnet, and the magnetic field was applied parallel to the direction of the light propagation. The transmitted light was detected by a photodiode with a lock-in amplifier.

### Sample Preparation

For the synchrotron XRD, XAS, and ESR measurements, ZnCoO powders were prepared using the sol-gel method^36, 40, 69^. For the synchrotron XRD and ESR measurements, 10% Co-doped ZnO powder was used, and for the synchrotron XAS measurement, 20% Co-doped ZnO powder was used. In the sol-gel process, Zn(CH_3_COOH)_2_·2H_2_O and Co(CH_3_COOH)_2_·4H_2_O were used as the starting materials. These were dissolved in CH_3_OCH_2_CH_2_OH(2-methoxyethanol) and stabilized by H_2_NCH_2_CH_2_OH (monoethanolamine). The dissolution and drying processes were performed in an argon (Ar) atmosphere and under vacuum conditions, respectively, to avoid external contamination. The residual organics were entirely eliminated by intermediate heat treatment at 300 °C and high crystallinity was ensured by a final heat treatment at 800 °C^40^.

For the MCD measurement, 10% Co-doped ZnO films were prepared by sputtering the ZnCoO target. The thin films were grown on an aluminum oxide (Al_2_O_3_) (0001) substrate at 300 °C in an atmosphere of Ar (99.999%). The working pressure was kept at 10 mTorr. This work was supported by Crystal Bank in Pusan National University.

### Hydrogen Injection

To suggest the consistency of hydrogen effect, we used two different hydrogen injection methods, which have been used in our previous studies. For the synchrotron XRD and MCD measurements, we used a plasma treatment for hydrogen injection, and different powers of plasma were used to achieve different hydrogen contents: 40 W (Hpla1), 60 W (Hpla2), and 80 W (Hpla3)^38, 40, 51, 52, 70^. In particular, H0 thin film was prepared by subjecting a pristine ZnCoO thin film to a hydrogen plasma treatment at 20 W in order to increase the carrier concentration of a pristine ZnCoO (~10^16^/cm^3^) and to rule out the effect of the increased carrier concentration by hydrogen treatment.

In the XAS experiment, we injected hydrogen using hot isostatic pressing (HIP-series)^39, 71–73^. The temperature was held constant at 300 °C, and the time and pressure were varied to obtain different hydrogen concentrations: HIP1 (500 bar and 1 hr), HIP2 (100 bar and 10 hr), and HIP3 (500 bar and 10 hr). The relative hydrogen content was determined by secondary ion mass spectrometry (SIMS) (IMS-6F; Cameca Co., Ltd., France).

### Theoretical Calculation

The theoretical density map was obtained from first principles electronic structure calculations based on the local-spin-density approximation and the Hubbard U (LSDA + U) method, with projector-augmented-wave pseudopotentials. These calculations were carried out by the Vienna ab initio Simulation Package (VASP)^30^.

## Electronic supplementary material

Supplementary information
