# Supplementary material for: Formation of ferromagnetic Co–H–Co complex and spin-polarized conduction band in Co-doped ZnO
Source: Sci Rep. 2017 Sep 11;7:11101. doi: 10.1038/s41598-017-11078-3 (PMC5593988; doi:10.1038/s41598-017-11078-3)
Supplement: Supplementary file 1 — Supplementary information [file 41598_2017_11078_MOESM1_ESM.pdf]

Supplementary Info.

Formation of ferromagnetic Co–H–Co complex and spin-polarized conduction band in Co-doped ZnO

Seunghun Lee[1,†], Ji Hun Park[2,†], Bum-Su Kim[2], Deok-Yong Cho[3], Yong Nam Choi[4], Tae-Woo Lee[5], Won-Kyung Kim[2], Doukyun Kim[6], Chae Ryong Cho[7], Chikako Moriyoshi[8], Chul Hong Park[9], Yoshihiro Kuroiwa[8,*], and Se-Young Jeong[2,*]

[1] Department of Materials Science and Engineering, University of Maryland, College Park, Maryland 20742, USA

[2] Department of Cogno-Mechatronics Engineering, Pusan National University, Miryang 50463, Republic of Korea

[3] IPIT and Department of Physics, Chonbuk National University, Jeonju 54896, Republic of Korea

[4] Neutron Science Division, Korea Atomic Energy Research Institute, Daejeon 34057, Republic of Korea

[5] KAIST Analysis center for Research Advancement, Daejeon 34141, Rep. Korea

[6] Department of Physics, Pusan National University, Busan 46241, Republic of Korea

[7] Department of Nano Fusion Technology, Pusan National University, Miryang 50463, Republic of Korea

[8] Department of Physical Science, Hiroshima University, Higashi-Hiroshima, 739-8526, Japan

[9] Department of Physical Education, Pusan National University, Busan 46241, Republic of Korea

[*] E-mail: syjeong@pusan.ac.kr

[*] E-mail: kuroiwa@sci.hiroshima-u.ac.jp

[†] S.L. and J.H.P. contributed equally to this work.

Oxygen defects in H-doped ZnCoO

Oxygen vacancies (V_O) are one type of crystal defect. They have been suspected to induce ferromagnetism in ZnCoO. To determine the number of V_O defects following hydrogen treatment, we calculated the oxygen occupancy based on the Rietveld refinement data obtained from the synchrotron radiation diffraction patterns. Table S1 shows the oxygen occupancies of ZnCoO samples with varying hydrogen content. The oxygen content of each sample is similar, considering the error bars. This indicates that the hydrogen treatment is not likely to induce V_O defects in ZnCoO, which suggests that the emergence of ferromagnetism after hydrogen injection is not due to the creation of V_O.[1,2]

Table S1. Change in oxygen occupancy of ZnCoO powders subjected to hydrogen plasma treatment (plasma-series), where the oxygen occupancy is obtained from X-ray diffraction and Rietveld refinement. The X-ray diffraction was measured at the BL02B2 beam-line of SPring-8.[3]

Sample label	Oxygen occupancy (error bar)
H0	1.01999 (0.00191)
Hpla1	1.02247 (0.00192)
Hpla2	1.01847 (0.00190)
Hpla3	1.02072 (0.00183)

Ferromagnetic resonance in H-doped ZnCoO

The observed ferromagnetic resonance (FMR) signal indicates that the Co spins have ferromagnetic spin interactions. The origin of the emergent FMR signal can be verified by analyzing *g*-factor. The *g*-factor of the observed FMR signal is ~ 3.3, which differs from the reported values for bulk metallic Co ($g = 2.18$)[4] and nano-sized Co clusters ($g_{hcp} = 8.48$, $g_{cubic} = 3.94$).[5] Therefore, the ferromagnetic signals observed in this work differ from those of the possible secondary phases, such as metallic Co precipitations. Accordingly, we can exclude metallic Co species as the origin of the observed FMR signal. This conclusion is in good agreement with the evidence from the synchrotron X-ray diffraction (XRD) and X-ray absorption experiments (XAS).

X-ray diffraction patterns and carrier concentration of ZnCoO thin films

Figure S1a shows the XRD patterns of ZnCoO thin films (plasma-series) used for the MCD measurement. The Bragg angles (2θ) at 34.4˚, 41.6˚, and 72.2˚ refer to the diffraction angles of ZnO (002), Al_2O_3 (0006), and ZnO (004), respectively. No peaks corresponding to secondary phases other than the ZnO wurtzite phase of the substrate are observed. No peak shift occurred, which indicates that the number of oxygen vacancies and zinc interstitials created following hydrogen treatment are negligible. Figure S1b shows the carrier concentrations (*n*) and motilities (*μ*) of ZnCoO thin films. As mentioned in the Methods section, the H0 thin film used in the MCD measurement was prepared by subjecting a pristine ZnCoO thin film to a hydrogen plasma treatment at 20 W to increase *n* of the pristine ZnCoO film. All thin films had similar *n* and *μ* values.

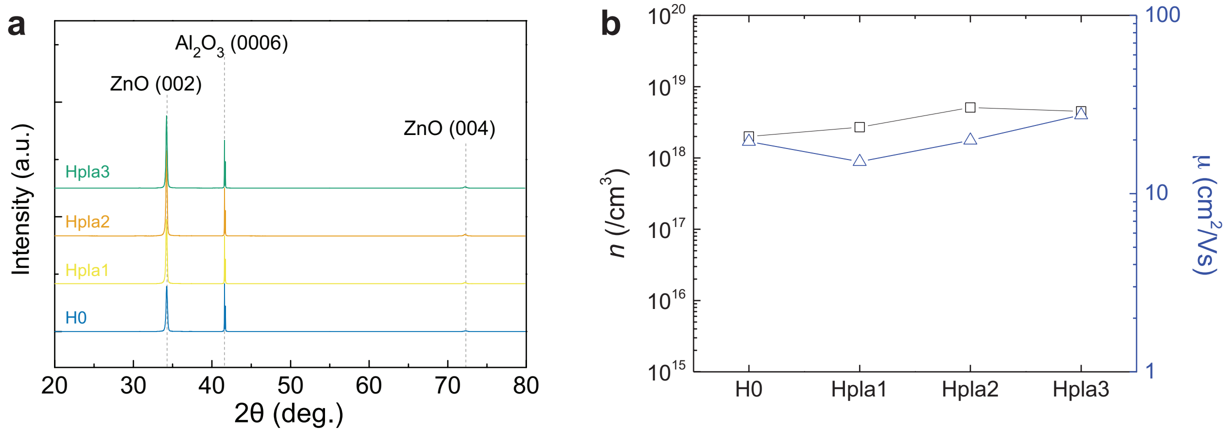

Figure S1. (a) X-ray diffraction patterns and (b) transport characteristics [*i.e.*, carrier concentration (*n*) and mobility (*μ*)] of ZnCoO thin films. The error bars of *n* and *μ* are less than 1%.

Optical absorption and band gap of ZnCoO thin films

Figure S2a shows the UV-vis absorption spectra of the ZnCoO thin films (plasma-series) used in the MCD measurement. All of the samples have similar absorption spectra, and the peaks (at 1.88, 2.02, and 2.18 eV) corresponding to the Co d-d* transition[6,7] are not significantly reduced or shifted after the hydrogen treatment. This indicates that the hydrogen treatment does not change the electrochemical state of the Co. The band gap of the ZnCoO thin films was determined using a Tauc plot (Fig. S2b) where the band gap energy can be obtained by extrapolating the linear region of $(\alpha h\upsilon)^2$. All of the thin films have the same band gap energy corresponding to 2.89±0.01 eV.

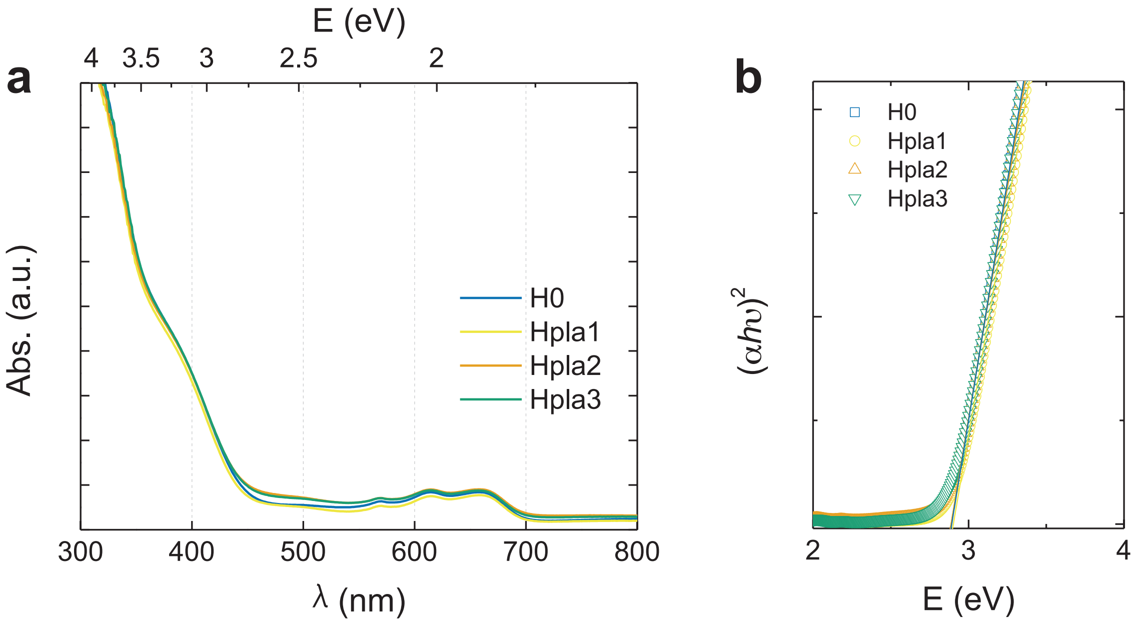

Figure S2. (a) UV-Vis absorption spectrum of ZnCoO thin films. (b) Tauc plot of ZnCoO thin films used to determine the direct band gap.

Theoretical density of states of Co-H-Co complexes

To theoretically support the spin polarization of the conduction band resulting from hydrogen incorporation, we performed theoretical calculations with two different methods: (i) DFT+Hubbard correction (DFT+U) and (ii) (i)+hybrid calculation (hybrid DFT+U). Firstly, we calculated the spin-dependent density of states (DOS) of a Co-Co pair without H, and a H-captured Co-Co pair (i.e., Co-H-Co)[8], based on DFT+U models. The first-principles calculations were performed by the Vienna simulation package (VASP) using the projector augmented-wave (PAW) method. We used the Perdew–Burke–Ernzerhof exchange-correlation functional (PBE) approach with the generalized gradient approximation (GGA) scheme, and the local spin density approximation (LSDA) method was used to compensate for the Coulomb interaction in the localized semi-core Zn-3d and Co-3d orbitals. We used U = 5 eV. As shown in Figure S3, there is a remarkable difference near the conduction band minimum between the two DOSs. This is caused by the formation of a localized state driven by the addition of H near the conduction band minimum. This model possibly elucidates the spin-dependent conduction band of hydrogen-incorporated ZnCoO. However, from a theoretical viewpoint, this model can be challenged by several limitations.[9] Even though the Hubbard correction (+U) is employed, the band gap of ZnO is still underestimated within standard DFT+U schemes. This band gap underestimation, moreover, can be linked to the energetically miscalculated states of the majority spin t_{2g} and minority spin e_g levels with respect to the valence band maximum (VBM) of ZnO.

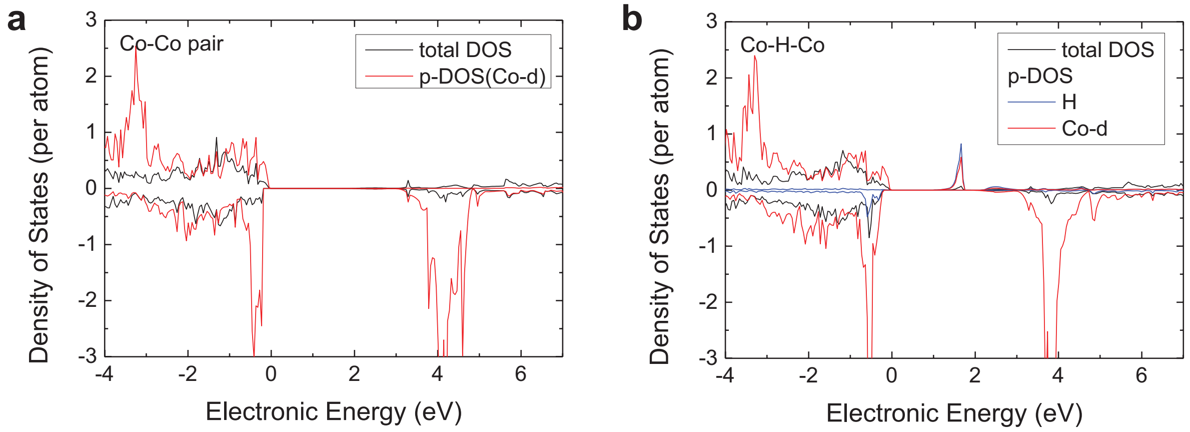

Figure S3. Through the DFT+U method, the calculated spin-dependent density of states (DOS) of (a) a Co-Co pair without H and (b) a H-captured Co-Co pair (i.e., Co-H-Co) are shown, where the partial DOS (p-DOS) for Co-3d and H are included. The positive and negative values indicate the majority and minority spin states, respectively.

Thus, we also performed the first-principles simulations based on the hybrid DFT+U calculations (Figure S4). The hybrid DFT+U is known to give the band gap close to the experimental value by combining the two calculations of the conventional DFT and the Hartree-Fock method. We used the HSE06 for the hybrid calculations with the parameter HFSCREEN =0.2.[10] Since the hybrid calculation does not treat the self-interaction error, which is significant for the localized orbital Zn-3d and Co-3d, we used the LDA+U method to include the effect of the Hubbard-U energy. The U values of the semicore Zn-3d and Co-3d orbitals were chosen to be the 7 eV and 5 eV, respectively. By the hybrid DFT+U calculations, the conduction band minimum (CBM) shifts upward to give a band gap of about 3.4 eV. However, the energy level of the local Co-H-Co orbital is not much shifted, as shown in Fig. S4. As a result, the Co-H-Co occupies a deep level below the CBM, and this level is electron-empty. In the presence of H impurities, the deep level of Co-H-Co can be occupied and possibly interact with a shallow donor type H level. It

accounts for the spin-dependent photon absorption observed in the MCD spectrum. Further theoretical studies are required to verify the contributions of hydrogen and Co-H-Co to the band structure of ZnCoO.

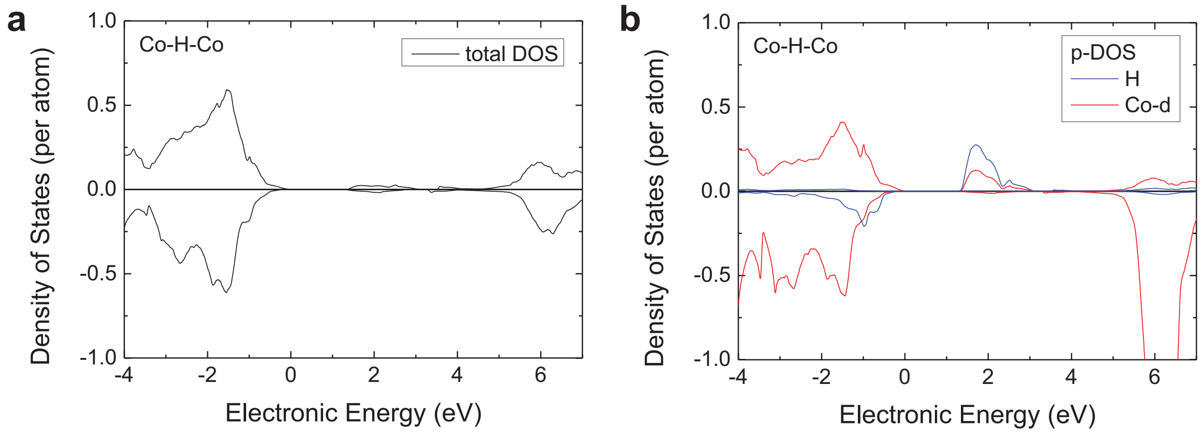

Figure S4. (a) Through the hybrid DFT+U method, the calculated total densities of electronic states of Co-H-Co complex and (b) the partial density of states of a Co atom and a H atom are shown. Since the spin of H is antiparallel to the spin of two Co atoms, and then the total magnetic moment of Co-H-Co is 5 μ_B. The U values of the semicore Zn-3d and Co-3d orbitals were chosen to be the 7 eV and 5 eV, respectively.

References

1. Park, J. H. *et al*. Analysis of oxygen vacancy in Co-doped ZnO using the electron density distribution obtained using MEM. *Nanoscale Res Lett* **10,** 657 (2015).
2. Park, J. H. *et al*. Effects of Al doping on the magnetic properties of ZnCoO and ZnCoO:H. *Appl. Phys. Lett*. **104**, 052412 (2014).
3. Nishibori, E., Takata, M., Kato, K. & Sakata, M. The large debye–scherrer camera installed at SPring-8 BL02B2 for charge density studies. *J. Phys. Chem. Sol*. **62**, 2095–2098 (2001).
4. Bardeleben, von, H. J., Jedrecy, N. & Cantin, J.-L. Ferromagnetic resonance signature of metallic Co clusters in ferromagnetic ZnCoO thin films. *Appl. Phys. Lett*. **93,** 142505 (2008).

5. Soboń, M. *et al*. FMR study of carbon coated cobalt nanoparticles dispersed in paraffin. *Reviews on Advanced Materials Science* **14,** 11–16 (2007).

6. Kim, S. J. *et al*. The comparison of the structural, magnetic, electronic, and optical properties for ZnCoO and Co-precipitation samples. *J. Korean Phys. Soc.* **56,** 1374-1377 (2010).

7. Schwartz, D. A., Norberg, N. S., Nguyen, Q. P., Parker, J. M. & Gamelin, D. R. Magnetic Quantum Dots: Synthesis, Spectroscopy, and Magnetism of Co^{2+}- and Ni^{2+}-Doped ZnO Nanocrystals. *J. Am. Chem. Soc.* **125,** 13205–13218 (2003).

8. Cho, Y. C. *et al*. Hydrogen-induced anomalous Hall effect in Co-doped ZnO. *New J. Phys.* **16,** 073030 (2014).

9. Walsh, A., Da Silva, J. L. F. & Wei, S.-H. Theoretical description of carrier mediated magnetism in Cobalt doped ZnO. *Phys. Rev. Lett.* **100**, 256401–256404 (2008).

10. Krukau, A. V., Vydrov, O. A., Izmaylov, A. F., & Scuseria, G. E., Influence of the exchange screening parameter on the performance of screened hybrid functionals. *J. Chem. Phys.* **125**, 224106 (2006).
